# Interactions of piRNAs with the mRNA of Candidate Genes in Esophageal Squamous Cell Carcinoma

**DOI:** 10.3390/cimb45070387

**Published:** 2023-07-23

**Authors:** Aizhan Rakhmetullina, Aigul Akimniyazova, Togzhan Niyazova, Anna Pyrkova, Makpal Tauassarova, Anatoliy Ivashchenko, Piotr Zielenkiewicz

**Affiliations:** 1 Institute of Biochemistry and Biophysics, Polish Academy of Sciences, 02-106 Warsaw, Poland; 2 Department of Technology of Production of Livestock Products, A. Baitursynov Kostanay Regional University, Kostanay 110000, Kazakhstan; 3 Higher School of Medicine, Faculty of Medicine and Healthcare, Al-Farabi Kazakh National University, Almaty 050040, Kazakhstan; 4 Faculty of Biology and Biotechnology, Al-Farabi Kazakh National University, Almaty 050040, Kazakhstan; 5 Center for Bioinformatics and Nanomedicine, Almaty 050060, Kazakhstan

**Keywords:** esophageal squamous cell carcinoma, gene, mRNA, piRNA, diagnosis

## Abstract

Recently, a database of human piRNAs (piwi-interacting RNAs) was created, which allows the study of the binding of many piRNAs to the mRNAs of genes involved in many diseases, including cancer. In the present work, we identified the piRNAs that can interact with candidate esophageal squamous cell carcinoma (ESCC) genes. The binding of 480 thousand piRNAs with the mRNAs of 66 candidate ESCC genes was studied. Bioinformatic studies found that piRNAs bind only to the mRNAs of nine candidate genes: *AURKA*, *BMP7*, *GCOM1*, *ERCC1*, *MTHFR*, *SASH1*, *SIX4*, *SULT1A1*, and *TP53*. It has been shown that piRNAs can bind to mRNA by overlapping nucleotide sequences in limited 3′UTR and 5′UTR regions called clusters of binding sites (BSs). The existence of clusters of piRNA BSs significantly reduces the proportion of the nucleotide sequences of these sites in the mRNA of target genes. Competition between piRNAs occurs for binding to the mRNA of target genes. Individual piRNAs and groups of piRNAs that have separate BSs and clusters of BSs in the mRNAs of two or more candidate genes have been identified in the mRNAs of these genes. This organization of piRNAs BSs indicates the interdependence of the expression of candidate genes through piRNAs. Significant differences in the ability of genes to interact with piRNAs prevent the side effects of piRNAs on genes with a lack of the ability to bind such piRNAs. Individual piRNAs and sets of piRNAs are proposed and recommended for the diagnosis and therapy of ESCC.

## 1. Introduction

After the sequencing of the human genome, methods of the molecular studies of the regulation of gene expression and the human genome began to develop. piRNAs (piwi-interacting RNAs) were identified more than twenty years ago [1,2]. In addition to these small RNAs, siRNAs (small interfering RNAs) capable of regulating gene expression were created [3,4]. For many years, due to a number of misconceptions, miRNAs and piRNAs were not widely used for practical purposes [5,6,7,8,9]. However, in vivo and in vitro experiments have successfully used siRNAs against undruggable targets for treating cancer and other diseases [10,11,12]. Therefore, candidate genes responsible for the development of many diseases have been identified [13,14,15,16,17].

This approach allows us to specify disease causes and perform diagnosis and develop therapies more effectively. The identification of candidate genes further requires the identification of factors regulating their expression. It has been shown that piRNAs found in human and animal cells are associated with gene expression, but it has not been established how these molecules can modify this process [6,17,18].

The asymptomatic nature of early esophageal cancer and late clinical manifestations result in poor prognosis and limited therapeutic success. Efforts to identify diagnostic/prognostic markers in clinics have not been successful. Thus, there is an urgent need to develop novel non-invasive biomarkers for the early diagnosis of esophageal cancer. Esophageal squamous cell carcinoma (ESCC) involves genes that encode regulators of numerous cellular processes, such as methylenetetrahydrofolate reductase (*MTHFR*), cell cycle control—(tumor protein p53 (*TP53*), Aurora kinase A (*AURKA*), bone morphogenetic protein-7 (*BMP7*), and others. Numerous genetic variations of these genes have been linked to ESCC during the past ten years using candidate gene approaches [19,20,21,22].

In previous work, we studied the piRNA interactions with mRNAs for esophageal adenocarcinoma, multiple sclerosis, and SARS-CoV-2 and found that these interactions can be evaluated by quantitative characteristics [23,24,25]. Many human genes are functionally related, so a single gene can affect several diseases. piRNA is a relatively new field of study, but recent findings have uncovered its crucial role in disease development. Specifically, piRNAs are involved in the regulation of gene expression, which can lead to the abnormal growth and spread of cancer cells [26]. Several publications have established piRNAs’ role in carcinogenesis [27,28,29]. An important fact is the possibility of preserving and transporting small RNA in the human body as part of exosomes and nanosized vesicles through the bloodstream [30,31,32,33]. After a database of piRNAs was created [34], it became possible to study the direct interaction of piRNAs with genes. The more than eight million piRNA molecules available in the database are difficult and expensive to study in wet experiments, so it is necessary to use bioinformatic approaches to identify the biological function of piRNAs more efficiently. These studies have shown the promise of this approach in determining the properties of piRNAs and their biological role. This work aimed to identify piRNAs that can interact with candidate esophageal squamous cell carcinoma (ESCC) genes and how this information could be used to diagnose the disease. 

## 2. Materials and Methods

The 66 candidate ESCC genes’ nucleotide sequences (Table S1, see Supplementary Materials) were downloaded from the National Center for Biotechnology Information (NCBI) website (accessed on 5 October 2022). The 480 thousand piRNAs’ nucleotide sequences were taken from Wang et al. [34]. The MirTarget program was used to predict the piRNA binding sites (BSs) in the mRNA [35]. The following characteristics of piRNA binding to mRNA are predicted by this program: the localization of the piRNA BSs in the 5′-untranslated region (5′UTR), coding domain sequence (CDS), and 3′-untranslated region (3′UTR) of the mRNAs; the schemes of nucleotide interactions between piRNAs and mRNA; and the free energy of the interaction between piRNAs and the mRNA (ΔG, kJ/mol); for each BS, the ratio ΔG/ΔGm (%) was calculated. The ΔGm equals the free energy of piRNA binding with its fully complementary canonical nucleotide sequence. The calculated data were used only to choose piRNAs whose nucleotides interacted with mRNA utilizing canonical (G-C and A-U) and noncanonical (G-U and A-C) nucleotides with a specific ΔG value. The MirTarget program studies the hydrogen bonds between piRNAs and mRNA based on the physicochemical properties of nucleotide interactions [35,36,37,38]. MirTarget differs from other programs in terms of finding piRNA BSs on mRNA in the following aspects: it takes into account the interaction of piRNA with mRNA over the entire piRNA sequence; it considers noncanonical G-U and A-C pairs; and it calculates the free energy of the interaction of the piRNAs with mRNA. It is notable that under identical circumstances, the G, A, C, and U nucleotides that make up the RNA structure of bacteria, plants, and mammals interact in the same way. Therefore, the physicochemical properties of canonical and noncanonical nucleotide pairs described above do not need additional proof of the previously established physicochemical characteristics of their interaction [36,37,38,39].

## 3. Results

In examining the interaction of piRNAs with mRNA genes, criteria were established for the following characteristics. The interaction of piRNAs and mRNA nucleotides must be along the entire length of the piRNAs. The free energy of the interaction must be at least 90% of the maximum ΔG/ΔGm. Splitting piRNAs into three groups based on the value of ΔG and ΔG/ΔGm of the interaction of piRNAs with mRNA will determine whether the values of ΔG and ΔG/ΔGm within the selected limits can reflect the basic properties of the interaction of piRNAs with mRNA. The property of candidate genes to interact with different numbers of piRNAs is acquired by selecting interrelated genes whose expression should be regulated by piRNAs as universal controllers of genome-wide expression. We determined whether piRNAs interact with specific individual mRNA sites and clarified the role of clusters of piRNAs BSs as a necessity for the coordinated regulation of gene expression with a requirement for compactness of gene regulatory sites. Bioinformatic technologies greatly facilitate the characterization of associations between piRNAs and mRNA interactions.

A study of 480 thousand piRNAs binding to the mRNAs of 66 candidate ESCC genes showed that the mRNAs of only nine genes could interact with piRNAs. The number of interacting piRNAs for each gene differed significantly. To elucidate the interaction of piRNAs with mRNAs, the interacting piRNAs were divided into three groups according to the free energy of interaction: piRNAs interacting with a ΔG value of −170 kJ/mol or higher (group 1), with a ΔG value of −160 kJ/mol to −169 kJ/mol (group 2), and with a ΔG value of −140 kJ/mol to −159 kJ/mol (group 3). This approach allowed us to clarify how the interacting groups of piRNAs may vary.

### 3.1. Characteristics of piRNAs’ Interaction with the mRNA of the AURKA Gene

**Figure 1:**
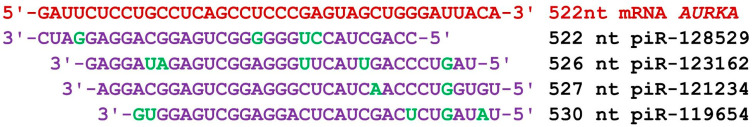
Nucleotide sequences of BSs of piRNAs and mRNA of the *AURKA* gene in the region from 522 nt to 560 nt. Note: The mRNA nucleotides are highlighted in red. Canonical pairs of piRNA and mRNA nucleotides are shown in violet, whereas noncanonical pairs are marked in green. The piRNAs’ names are followed by the start of their BSs.

The mRNA of the *AURKA* gene consists of 566 nt of 5′UTR, 1212 nt of CDS, and 770 nt of 3′UTR regions. The start of the piRNAs’ group 1 BSs is localized only in the 5′UTR from 410 nt to 533 nt (Table S2). The BSs of 43 piRNAs were located with overlapping nucleotide sequences, that is, they formed a cluster of BSs and were localized at the end of the 5′UTR. The BSs’ cluster was 157 nt long and contained BS piRNAs with lengths of 33 nt and 34 nt. Group 2 consisted of 38 piRNAs whose BSs were localized in the BS cluster starting from 414 nt to 534 nt with a length of 153 nt (Table S2). The piRNAs of group 2 varied in length from 31 nt to 34 nt. In this cluster of BSs also located in the 5′UTR, 38 piRNAs from piR-49581 to piR-363915 were bound. The piRNAs from group 3 were from 27 nt to 33 nt in length. Group 3 consisted of 60 piRNAs whose BSs were localized in a cluster of BSs (410 nt to 536 nt with a length of 156 nt). Consequently, this cluster of BSs also ended at the 5′UTR before the CDS. The piRNAs from this group were 27 nt to 33 nt long. Group 3 included piRNAs in the range from piR-30739 to piR-480279. The BSs of 141 piRNAs of the three groups were in a cluster 156 nt long with 1.1 nt per piRNA, indicating a high density of BSs for piRNAs. Figure 1 shows the nucleotide sequences of four piRNAs that interacted with a cluster of BSs located in the 5′UTR mRNA of the *AURKA* gene with overlapping nucleotide sequences. From these data, we can see how piRNAs will compete for binding in the BS cluster.

### 3.2. Characteristics of piRNAs’ Interaction with the mRNA of the BMP7 Gene

**Figure 2:**
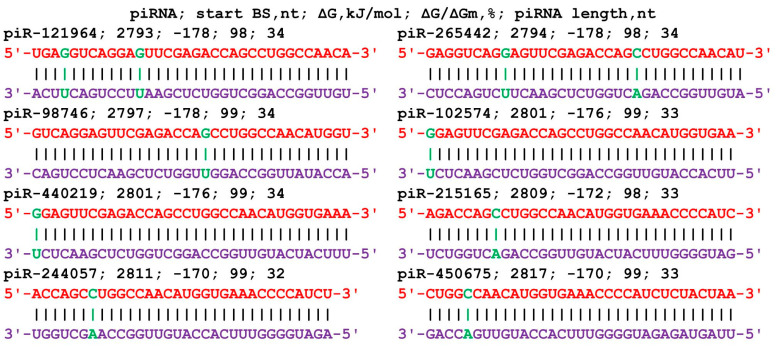
Nucleotide sequences of piRNAs and the characteristics of their interaction with the mRNA of the *BMP7* gene. The mRNA nucleotides are highlighted in red. Canonical pairs of piRNA and mRNA nucleotides are shown in violet, whereas noncanonical pairs are marked in green.

The mRNA of the *BMP7* gene includes a 5′UTR of 529 nt, a CDS of 1296 nt, and a 3′UTR of 2207 nt. piRNAs from all three groups bound only in the 3′UTR (Table S3). In group 1, the BSs of 40 piRNAs ranged from 2756 nt to 2889 nt, and the length of the BS cluster was 166 nt. The length of the binding piRNAs was 33 nt and 34 nt. Forty piRNAs from piR-43404 to piR-440219 were bound in the cluster. Within the large cluster was a 56 nt BS cluster for eight piRNAs with a ΔG/ΔGm value of 98% to 99% and a free energy value of −170 kJ/mol to −178 kJ/mol (Figure 2). This corresponds to the formation of predominantly canonical nucleotide pairs except for two noncanonical pairs in the interaction of piR-121964 and piR-265442 and one noncanonical pair in each of the remaining piRNAs. Group 2 included 67 piRNAs of length 31 nt to 34 nt that bound in a cluster (2729 nt to 2970 nt with length 273 nt). Group 3 included 111 piRNAs 28–34 nt long; the beginning of the BSs was located from 2733 nt to 2981 nt, and the BS cluster length was 279 nt. The BSs of the piRNAs were unevenly distributed in the clusters, with a total of 1.3 nt per piRNA in the cluster from 2729 nt to 3012 nt for 218 piRNAs of the three groups.

### 3.3. Characteristics of piRNAs’ Interaction with the mRNA of the ERCC1 Gene

**Figure 3:**
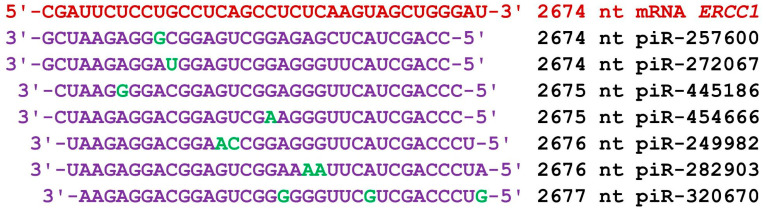
Nucleotide sequences of the BSs of piRNAs and the mRNA of the *ERCC1* gene in the region from 2674 nt to 2710 nt. Note: The mRNA nucleotides are highlighted in red. Canonical pairs of piRNA and mRNA nucleotides are shown in violet, whereas noncanonical pairs are marked in green. The piRNAs’ names are followed by the start of their BSs.

The mRNA of the *ERCC1* gene consists of 146 nt of 5′UTR, 822 nt of CDS, and 2343 nt of 3′UTR in terms of length. The start of the BSs of 47 piRNAs of group 1 with ΔG values varying from −170 kJ/mol to −180 kJ/mol was located in the 3′UTR from 2597 nt to 2794 nt (a cluster with a length of 228 nt; Table S4). The length of the piRNAs varied from 32 nt to 34 nt. Forty-nine piRNAs of group 2 bound to the mRNA. The BSs of these piRNAs were also localized in the 3′UTR from 2592 nt to 2796 nt. The BS cluster of 49 piRNAs was 235 nt long, accounting for 10% of the 3′UTR length; in this cluster of BSs, piRNAs with numbers from piR-41630 to piR-365910 bound. The beginning of piRNAs’ BSs in group 3 was 2595 nt to 2785 nt, and the length of the cluster of BSs was 221 nt. Consequently, the clusters of BSs of the three groups of piRNAs were close, and each of the 158 piRNAs averaged 1.5 nt. piR-289349, piR-298414, and piR-436360 bound with mRNA of the *ERCC1* gene fully complementary, forming canonical nucleotide pairs only, and the ΔG/ΔG value was 100% (Table S4). An example of a very compact arrangement of piRNAs’ BSs to the mRNA of the ERCC1 gene is shown in Figure 3. In the 35 nt cluster, seven piRNAs were bound predominantly by canonical nucleotide pairs.

### 3.4. Characteristics of piRNAs’ Interaction with the mRNA of the GCOM1 Gene

**Figure 4:**
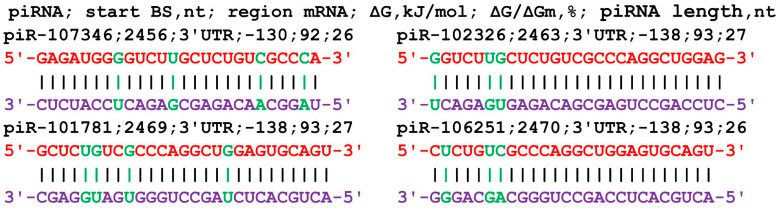
Nucleotide sequences of piRNAs and characteristics of their interaction with the mRNA of the *GCOM1* gene. The mRNA nucleotides are highlighted in red. Canonical pairs of piRNA and mRNA nucleotides are shown in violet, whereas noncanonical pairs are marked in green.

The nucleotide sequence of the *GCOM1* gene consists of 131 nt of 5′UTR, 1653 nt of CDS, and 2879 nt of 3′UTR in terms of length. An analysis of the interaction parameters of the 16 piRNAs of group 1 with the mRNA of the *GCOM1* gene shows that these piRNAs bind from 2458 nt to 2466 nt in the 3′UTR only (Table S5). The beginning of the BS cluster of these piRNAs with a length of 33–34 nt begins at 2458 nt and ends at position 2499 nt, i.e., its length is 41 nt. This cluster of BSs binds piRNAs with numbers from piR-56610 to piR-465348. All of the starts of the BSs of the 20 piRNAs of group 2 are localized by mRNA in the site from 2453 nt to 2486 nt in the 3′UTR with a BS cluster length of 66 nt. In this cluster of BSs, piRNAs with numbers from piR-34813 to piR-458324 bind. The length of the interacting piRNAs varied from 30 to 34 nt. The 14 piRNAs of group 3 bound from 2456 nt to 2488 nt in a cluster with a length of 61 nt. In this cluster of BSs, piRNAs bind with numbers ranging from piR-117518 to piR-468536. On average, 1.3 nt of the 50 piRNAs interact with the mRNA of the *GCOM1* gene in the 66 nt cluster. piRNAs less than 30 nt in length can also interact with the *GCOM1* gene (Figure 4). The ΔG/ΔGm value was higher than 92%, indicating the preferential formation of canonical nucleotide pairs.

### 3.5. Characteristics of piRNAs’ Interaction with the mRNA of the MTHFR Gene

**Figure 5:**
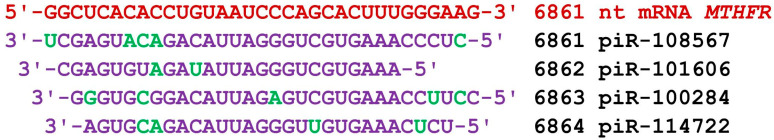
Nucleotide sequences of the BSs of piRNAs and the mRNA of the *MTHFR* gene in the region from 6861 nt to 6893 nt. Note: The mRNA nucleotides are highlighted in red. Canonical pairs of piRNA and mRNA nucleotides are shown in violet, whereas noncanonical pairs are marked in green. The piRNAs’ names are followed by the start of their BSs.

The mRNA of the *MTHFR* gene has a long 3′UTR of 4951 nt, unlike the mRNA of the other candidate ESCC genes studied. The 5′UTR and CDS consist of 229 nt and 1971 nt, respectively. Group 1 piRNAs bound to the 3′UTR in two clusters of BSs (Table S6). The first cluster of BSs contained BSs from 6182 nt to 6385 nt, and the second from 6849 nt to 7051 nt. The distance between the second cluster’s beginning and the first cluster’s end was 464 nt. Accordingly, 16 and 29 piRNAs were bound in these clusters of BSs. The length of both clusters was 235 nt. The first cluster bound piRNAs from piR-47672 to piR-456736 and the second cluster from piR-36604 to piR-475473. Group 2 piRNAs also bound in two clusters of BSs: the beginning BSs of the first cluster were from 6190 nt to 6383 nt, and the second cluster was from 6849 nt to 7062 nt. The interval between the end and beginning of the clusters was 466 nt. In the first and second clusters, 12 and 53 piRNAs were bound, respectively. Group 3 piRNAs also bound in two clusters of BSs (Table S6). The first cluster of BSs began at 6177 nt and ended with piRNAs binding at the 6388 nt position. It bound 25 piRNAs, and the second cluster bound 78 piRNAs. The second cluster started at 6848 nt, and the last piRNAs were bound at position 7056 nt. As a result, 53 piRNAs of the three groups were bound in the first cluster and 160 piRNAs were bound in the second cluster. The total length of the first cluster for the three groups of piRNAs was 240 nt and that of the second cluster 246 nt. As a result, the first cluster had 4.5 nt per piRNAs and the second cluster had 1.5 nt. Both clusters of BSs were localized at the end of the 3′UTR mRNA of the *MTHFR* gene. Figure 5 shows one of several clusters of BSs formed from the BSs of four competing piRNAs located one to three nucleotides apart.

### 3.6. Characteristics of piRNAs’ Interaction with the mRNA of the SASH1 Gene

**Figure 6:**
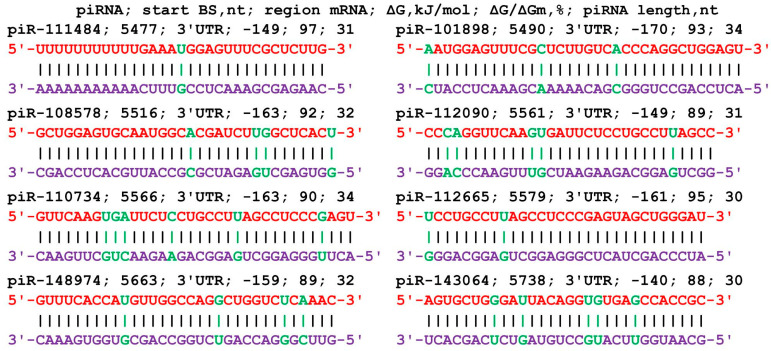
Nucleotide sequences of piRNAs and the characteristics of their interaction with the mRNA of the *SASH1* gene. The mRNA nucleotides are highlighted in red. Canonical pairs of piRNA and mRNA nucleotides are shown in violet, whereas noncanonical pairs are marked in green.

The mRNA of the *SASH1* gene in the 5′UTR, CDS, and 3′UTR contained 475 nt, 3744 nt, and 3491 nt, respectively. The 63 piRNAs of group 1 had the beginnings of BSs only in the 3′UTR from 5490 nt to 5741 nt (Table S7). The piRNA interval from piR-26543 to piR-459543 indicates the participation of piRNAs from the entire studied range of 480 thousand piRNAs. The 61 piRNAs in group 2 had the beginnings of BSs in the 3′UTR from 5507 nt to 5747 nt. The piRNAs from piR-40007 to piR-355919 were associated with this cluster. The piR-50295 that bound to the mRNA of the *SASH1* gene is fully complementary with a ΔG/ΔG value of 100%. The 73 piRNAs of group 3 had the beginnings of BS in the 3′UTR from 5491 nt to 5741 nt. In this cluster, piRNAs from piR-21000 to piR-466023 were bound. The length of the cluster common to the three groups of piRNAs from 5490 nt to 5777 nt was 287 nt in which 197 piRNAs were bound, hence 1.5 nt per piRNAs. Figure 6 shows diagrams of the interaction of several piRNAs with the mRNA of the *SASH1* gene, indicating their binding using canonical and noncanonical nucleotide pairs.

### 3.7. Characteristics of piRNAs’ Interaction with the mRNA of the SIX4 Gene

**Figure 7:**
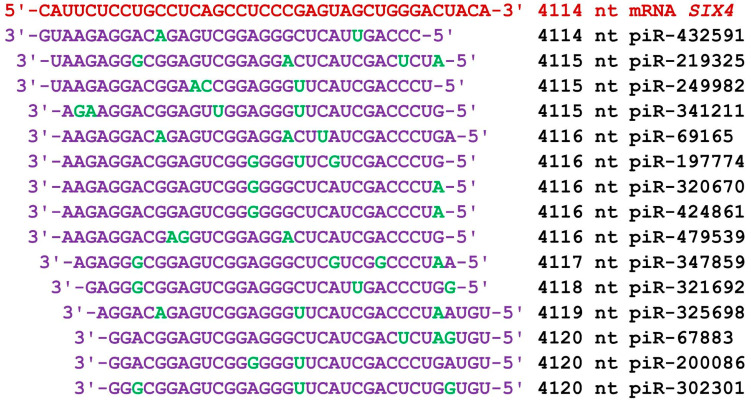
Nucleotide sequences of the BS cluster in the 3′UTR of the mRNA of the *SIX4* gene from 4114 nt and the nucleotide sequences of 15 piRNAs. Note: The mRNA nucleotides are highlighted in red. Canonical pairs of piRNA and mRNA nucleotides are shown in violet, whereas noncanonical pairs are marked in green. The piRNAs’ names are followed by the start of their BSs.

The mRNAs of the *SIX4* gene in the 5′UTR, CDS, and 3′UTR contained 60 nt, 2346 nt, and 3870 nt, respectively. There were 86 piRNAs in group 1 whose beginning of the BSs was located only in the 3′UTR from 4026 nt to 4266 nt (Table S8). In this interval, piRNAs from piR-38669 to piR-479539 bound to mRNAs. The 83 piRNAs of group 2 bound from 4021 nt to 4259 nt. The corresponding piRNAs were between piR-40921 and piR-414045. There were 105 piRNAs in group 3 with the beginning of their BSs also located in the 3′UTR from 4013 nt to 4269 nt. The length of this BS cluster was 287 nt. The 105 piRNAs included piR-20572 to piR-480249. The number of the three groups of piRNAs was 274, which were bound in a 287 nt cluster with a high density of 1.1 nt per piRNA. Figure 7 shows the nucleotide sequences of the piRNAs and the mRNA of the *SIX4* gene in one of the clusters, clearly indicating a fully complementary interaction between their nucleotides. These results clearly indicate high competition among 15 piRNAs for binding in this cluster. If any piRNA is present in concentrations an order of magnitude higher than the others, it will dominate in the suppression of mRNA translation.

### 3.8. Characteristics of piRNAs’ Interaction with the mRNA of the SULT1A1 Gene 

**Figure 8:**
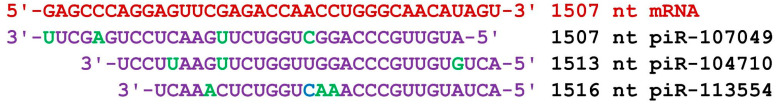
Nucleotide sequences of the BSs of piRNAs and the mRNA of the *SULT1A1* gene in the region from 1507 nt to 1543 nt. Note: The mRNA nucleotides are highlighted in red. Canonical pairs of piRNA and mRNA nucleotides are shown in violet, whereas noncanonical pairs are marked in green. The piRNAs’ names are followed by the start of their BSs.

The nucleotide sequence of the *SULT1A1* gene consisted of 473 nt 5′UTR, 888 nt CDS, and 231 nt 3′UTR in terms of length. An analysis of the interaction of the eight piRNAs from group 1 with the mRNA of the *SULT1A1* gene shows that these piRNAs started binding from 1483 nt to 1510 nt only in the 3′UTR (Table S9). In this cluster of BSs, piRNAs with numbers ranging from piR-10749 to piR-475473 bound. All of the beginnings of ten piRNAs BSs from group 2 were localized in the mRNA at the 1482 nt to 1521 nt site in the 3′UTR with a BS cluster length of 72 nt. In this cluster of BSs, piRNAs with numbers from piR-200742 to piR-472871 bound. The 11 piRNAs of group 3 were bound from 1489 nt to 1517 nt in a cluster with a length of 69 nt. In this cluster of BSs, piRNAs with numbers ranging from piR-104710 to piR-447258 bound. On average, one of the 29 piRNAs interacting with the mRNA of the *SULT1A1* gene in the 71 nt cluster was 2.4 nt. The BS cluster of all piRNAs was located at the end of the 3′UTR. The small number of piRNAs that bound to the mRNA of the *SULT1A1* gene is probably due to the short length of the 3′UTR. The low density of piRNAs’ BSs in the mRNA of the gene revealed only one cluster for the binding of three piRNAs, with a significant shift in the beginning of the BSs (Figure 8).

### 3.9. Characteristics of piRNAs’ Interaction with the mRNA of the TP53 Gene

**Figure 9:**
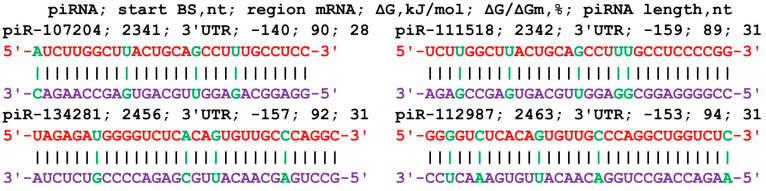
Nucleotide sequences of piRNAs and the characteristics of their interaction with the mRNA of the *TP53* gene. Note: The mRNA nucleotides are highlighted in red. Canonical pairs of piRNA and mRNA nucleotides are shown in violet, whereas noncanonical pairs are marked in green.

The mRNAs of the *TP53* gene mRNAs in the 5′UTR, CDS, and 3′UTR contained 197 nt, 1041 nt, and 1409 nt, respectively. In group 1, there were 19 piRNAs whose beginning BSs were located only in the 3′UTR from 2293 nt to 2512 nt (Table S10). In this interval, mRNAs bound piRNAs from piR-44059 to piR-455123. The 30 piRNAs of group 2 bound from 2292 nt to 2516 nt. The corresponding piRNAs were between piR-40367 and piR-291404. Group 3 had 42 piRNAs beginning with their BSs in the 3′UTR from 2290 nt to 2533 nt. The 42 piRNAs included piR-32165 through piR-365438. The number of piRNAs of the three groups was 91, bound in a cluster 274 nt long with a density of 3.0 nt per piRNA. It should be noted that there were portions of the cluster that did not bind piRNAs between 2301 nt and 2382 nt (length 81 nt) and between 2390 nt and 2456 nt (length 66 nt); the cluster was reduced by 147 nt, and the working part of the cluster was 127 nt with 1.4 nt per piRNA, which is comparable to the results obtained for other candidate genes. Two BSs at 163 nt intervals of ten piRNAs binding at positions 2293 nt and 2456 nt were identified in the mRNA of the *TP53* gene. These are piR-101480 and piR-69443 from group 1 piRNAs; piR-106322, piR-53597, and piR-62868 from group 2 piRNAs; and piR-32165, piR-37715, piR-52128, piR-57508, and piR-32194 from group 3. This is explained by the presence of two sites in the 3′UTR mRNA of the *TP53* gene with identical nucleotide sequences binding these piRNAs. The relatively small number of piRNAs interacting with the mRNA of the gene (Table S10) is reflected in the absence of large clusters of BSs in the mRNA of the *TP53* gene. However, the binding of piRNAs to the mRNA was efficient (Figure 9).

### 3.10. piRNAs That Bind to the mRNA of Two or More Candidate Genes

It is essential to know which piRNAs bind to which genes and how this knowledge can be adequately used to regulate gene expression with piRNAs. We analyzed the interaction of piRNAs in nine candidate ESCC genes. Finding BSs of piRNAs in the mRNAs of alternative candidate genes was considered positive when the ΔG value was −160 kJ/mol or higher and the ΔG/ΔGm value was 90% or higher. 

**Figure 10:**
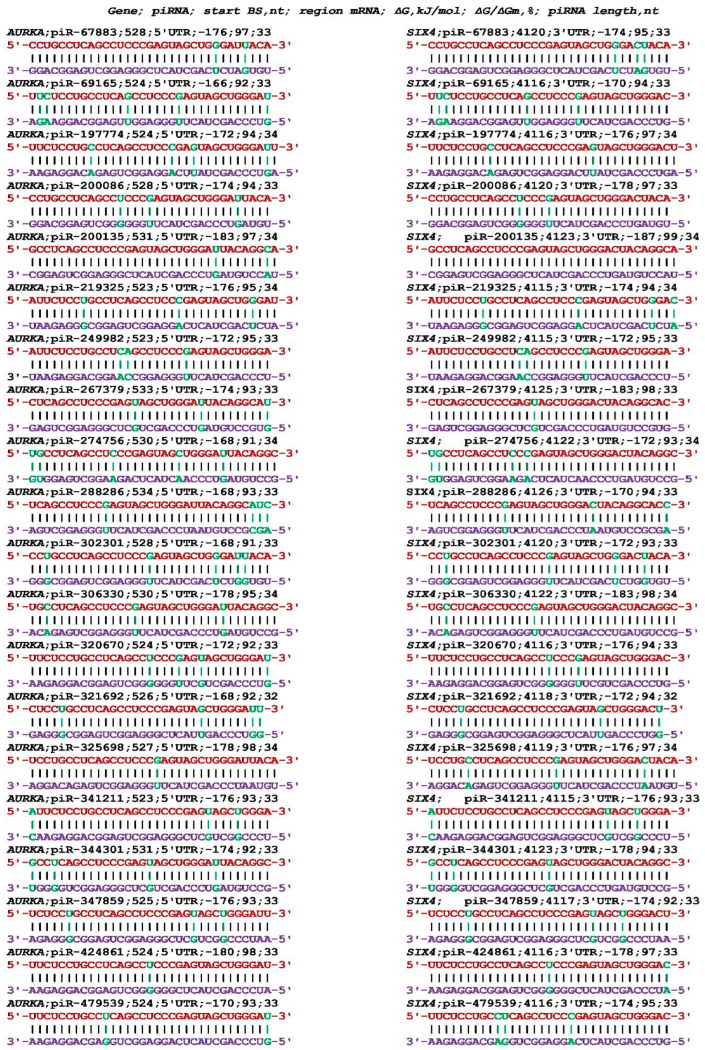
Schemes of the interaction of identical piRNAs with the mRNAs of the *AURKA* and *SIX4* genes. Note: The mRNA nucleotides are highlighted in red. Canonical pairs of piRNA and mRNA nucleotides are shown in violet, whereas noncanonical pairs are marked in green.

Table S11 shows the search results for piRNAs that bind to the mRNAs of the *AURKA*, *ERCC1*, *SASH1*, and *SIX4* genes. Only piR-55670 and piR-93385 bound to the mRNA of the *AURKA* and *BMP7* genes. Only piR-55670, piR-93385, piR-89432, piR-218175, piR-55670, and piR-93385 bound to the mRNA of the *AURKA* and *MTHFR* genes. The piR-67883, piR-200086, and piR-306330 bound to the mRNAs of the *AURKA* and *TP53* genes. An example of the interaction between the piRNAs in the *AURKA* and *SIX4* genes is shown in Figure 10.

The piRNAs interacting with the mRNAs of the *AURKA* and *SIX4* genes bound in the clusters of BSs with similar nucleotide sequences in the mRNA of both genes. In the mRNA of the *AURKA* gene, piRNAs bound in the 5′UTR from 523 nt to 534 nt, and in the mRNA of the *SIX4* gene, piRNAs bound in the 3′UTR from 4115 nt to 4126 nt with the same difference in BS beginning at about 11 nt. The value of the free binding energy of each piRNA in both mRNAs was close and high. The results demonstrate that one of the functions of the 5′UTR and 3′UTR is the binding of piRNAs regulating mRNA translation.

**Figure 11:**
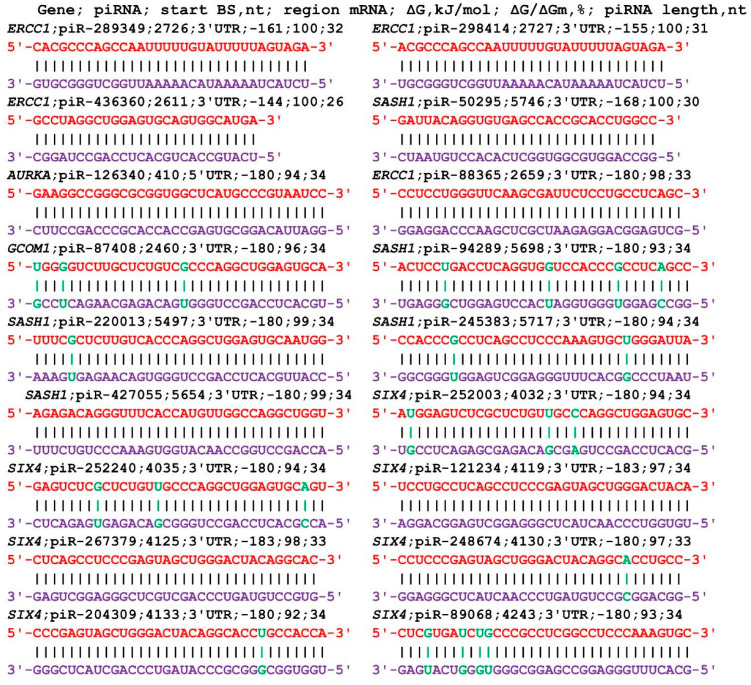
Schemes of the interaction between piRNAs and the mRNAs of candidate genes with ΔG/ΔGm equal to 92% to 100% and a ΔG value equal to -180 kJ/mol and higher. Canonical pairs of piRNA and mRNA nucleotides are shown in violet, whereas noncanonical pairs are marked in green.

As a result, the mRNAs of all candidate genes binding piRNAs had BSs in two or more mRNAs. These data indicate the interconnection of candidate genes through the interaction of piRNAs, which should be considered both in developing diagnostic methods for ESCC and in creating therapeutic drugs using piRNAs. For using piRNAs as markers of ESCC, it is logical to use the genes that are targeted with the highest free energy of interaction between their mRNA and piRNAs, as well as the genes whose mRNAs bind mainly due to canonical nucleotide pairs. Figure 11 shows the interaction schemes of such associations of piRNAs and mRNA candidate genes. piR-289349, piR-298414, and piR-436360 can completely block protein synthesis at a concentration equal to or greater than that of the mRNA of *ERCC1*. Similarly, piR-50295 can completely inhibit the translation of the mRNA of the *SASH1* gene.

## 4. Discussion

The free energy value of the interaction of piRNAs with mRNA determines the probability of their interaction with mRNA. Depending on the free energy of interaction of piRNAs with the mRNA of a gene, groups of piRNAs differ little in the BS interval and their position in the mRNA region, which is due to the selection during the evolution of the site for binding piRNAs with different lengths and nucleotide compositions. Therefore, changing piRNAs’ length and nucleotide composition leads to the selection of optimal piRNAs for the desired effect in regulating the expression of one or more genes. The piRNAs that we identified regulating two or more candidate genes formed both single bonds in regulating the expression of these genes and associations of several genes regulated by a group of piRNAs. When studying the interaction of piRNAs with mRNA genes, criteria were established for the following characteristics. 1. The piRNAs’ interaction with the mRNAs of candidate ESCC genes occurs along the entire length of the piRNAs. 2. The free interaction energy (ΔG) must be at least 90% of the maximum ΔGm value. 3. The division of piRNAs into three groups according to the value of ΔG and ΔG/ΔGm of the interaction of piRNAs with mRNA allowed us to determine that these characteristics do not fundamentally change the basic properties of the interaction of piRNAs with mRNA within the chosen limits. 

The above results indicate non-random binding of piRNAs in a narrow interval of mRNA length only in the 3′UTR. This allows the human genome to control gene expression by many piRNAs, keeping only a small portion of the gene conserved. Another property of piRNAs is the presence of BSs of some piRNAs in the mRNA of more than one gene. This fact indicates the general dependence of a group of candidate genes on the regulatory influence of the same piRNAs. This important property must be known for each piRNA when using piRNAs as disease markers and therapeutic agents. Ignorance of this property can lead to side effects when using piRNAs. 

The organization of BSs into clusters reduces the mRNA region responsible for regulating expression by piRNA molecules, which must be kept conservative to preserve the regulation of genome expression balanced during evolution. The binding of piRNAs in clusters leads to competition between them for the ability to exert a regulatory effect on the expression of candidate genes. At the same time, the competition between piRNAs ensures the stability of their regulation of the target gene expression because an increase or decrease in the concentration of any piRNA will have a compensatory effect on other piRNAs. That is, a significant effect of one piRNA requires considerable increases in its concentration to exceed the total impact of other piRNAs. 

Using piRNAs for disease diagnosis requires compliance with several criteria that will ensure the validity of the proposed piRNAs as markers. These are as follows: 1. piRNAs must bind to mRNAs with sufficiently high free energy to affect the expression of one or more candidate genes; 2. piRNAs must be expressed in a manner that is ordinarily comparable to the expression of candidate target genes; 3. Measurements of piRNAs and candidate gene concentrations should be made simultaneously, directly indicating which piRNAs correlate with the target gene’s expression level. The studies conducted show specific associations of piRNAs and target genes that need to be studied. The findings contribute to determining the expression level of piRNAs and their target genes under normal and disease conditions for the diagnosis and therapy of diseases.

## Data Availability

Not applicable.

## Supplementary Materials

The following supporting information can be downloaded at https://www.mdpi.com/article/10.3390/cimb45070387/s1, Table S1: List of candidate genes for ESCC; Table S2: Characteristics of the piRNA interaction with the mRNA of the *AURKA* gene; Table S3: Characteristics of the piRNA interaction with the mRNA of the *BMP7* gene; Table S4: Characteristics of the piRNA interaction with the mRNA of the *ERCC1* gene; Table S5: Characteristics of the piRNA interaction with the mRNA of the *GCOM1* gene; Table S6: Characteristics of the piRNA interaction with the mRNA of the *MTHFR* gene; Table S7: Characteristics of the piRNA interaction with the mRNA of the *SASH1* gene; Table S8: Characteristics of the piRNA interaction with the mRNA of the *SIX4* gene; Table S9: Characteristics of the piRNA interaction with the mRNA of the *SULT1A1* gene; Table S10: Characteristics of the piRNA interaction with the mRNA of the *TP53* gene; Table S11: piRNAs that bind to the mRNA of the *AURKA*, *ERCC1*, *SASH1* and *SIX4* genes.

## References

[1] Kim V.N. (2006). Small RNAs just got bigger: Piwi-interacting RNAs (piRNAs) in mammalian testes. Genes Dev..

[2] Aravin A.A., Sachidanandam R., Girard A., Fejes-Toth K., Hannon G.J. (2007). Developmentally regulated piRNA clusters implicate MILI in transposon control. Science.

[3] Fire A., Xu S., Montgomery M.K., Kostas S.A., Driver S.E., Mello C.C. (1998). Potent and specific genetic interference by double-stranded RNA in Caenorhabditis elegans. Nature.

[4] Timmons L., Tabara H., Mello C.C., Fire A.Z. (2003). Inducible Systemic RNA Silencing in *Caenorhabditis elegans*. Mol. Biol. Cell.

[5] Singh G., Mallick B. (2022). Predicting sequence and structural features of effective piRNA target binding sites. J. Mol. Recognit..

[6] Xiong Q., Zhang Y., Li J., Zhu Q. (2022). Small Non-Coding RNAs in Human Cancer. Genes.

[7] Wang X., Gou L.T., Liu M.F. (2022). Noncanonical functions of PIWIL1/piRNAs in animal male germ cells and human diseases. Biol. Reprod..

[8] Zhang J., Chen S., Liu K. (2022). Structural insights into piRNA biogenesis. Biochim. Biophys. Acta Gene Regul. Mech..

[9] Priyadarshini M., AlHarbi S., Frøkjær-Jensen C. (2022). Acute and inherited piRNA-mediated silencing in a rde-3 ribonucleotidyltransferase mutant. microPubl. Biol..

[10] Feng Y., Lin Y., Jiang Z., Wu L., Zhang Y., Wu H., Yuan X. (2023). Insulin-like growth factor-2 mRNA-binding protein 3 promotes cell migration, invasion, and epithelial-mesenchymal transition of esophageal squamous cell carcinoma cells by targeting zinc finger E-box-binding homeobox 1 mRNA. Mol. Carcinog..

[11] Sinha A., Bhattacharjee R., Bhattacharya B., Nandi A., Shekhar R., Jana A., Saha K., Kumar L., Patro S., Panda P.K. (2023). The paradigm of miRNA and siRNA influence in Oral-biome. Biomed. Pharmacother..

[12] Lee Y.R., Tsai H.P., Yeh C.S., Fang C.Y., Chan M.W.Y., Wu T.Y., Shen C.H. (2022). RNA Interference Approach Is a Good Strategy against SARS-CoV-2. Viruses.

[13] Chattopadhyay T., Gupta P., Nayak R., Mallick B. (2023). Genome-wide profiling of dysregulated piRNAs and their target genes implicated in oncogenicity of tongue squamous cell carcinoma. Gene.

[14] Belkozhayev A., Niyazova R., Wilson C., Jainakbayev N., Pyrkova A., Ashirbekov Y., Akimniyazova A., Sharipov K., Ivashchenko A. (2022). Bioinformatics Analysis of the Interaction of miRNAs and piRNAs with Human mRNA Genes Having di- and Trinucleotide Repeats. Genes.

[15] AmeliMojarad M., Amelimojarad M. (2022). piRNAs and PIWI proteins as potential biomarkers in Breast cancer. Mol. Biol. Rep..

[16] Ray S.K., Mukherjee S. (2022). Piwi-interacting RNAs (piRNAs) and colorectal carcinoma: Emerging non-invasive diagnostic biomarkers with potential therapeutic target based clinical implications. Curr. Mol. Med..

[17] Riquelme I., Pérez-Moreno P., Letelier P., Brebi P., Roa J.C. (2021). The Emerging Role of PIWI-Interacting RNAs (piRNAs) in Gastrointestinal Cancers: An Updated Perspective. Cancers.

[18] Heng B., Xie X., Zeng W., Li H., Shi L., Ye W., Wu F. (2022). PIWI-Interacting RNA Pathway Genes: Potential Biomarkers for Clear Cell Renal Cell Carcinoma. Dis. Markers.

[19] Urabe Y., Kagemoto K., Hayes C.N., Nakamura K., Masuda K., Ono A., Tanaka S., Arihiro K., Chayama K. (2019). Genomic characterization of early-stage esophageal squamous cell carcinoma in a Japanese population. Oncotarget.

[20] Chen X.X., Zhong Q., Liu Y., Yan S.M., Chen Z.H., Jin S.Z., Xia T.L., Li R.Y., Zhou A.J., Su Z. (2017). Genomic comparison of esophageal squamous cell carcinoma and its precursor lesions by multi-region whole-exome sequencing. Nat. Commun..

[21] Xu G., Tang S., Yang J., Chen K., Kang J., Zhao G., Feng F., Yang X., Zhao L., Lu Q. (2013). BMP7 expression in esophageal squamous cell carcinoma and its potential role in modulating metastasis. Dig. Dis. Sci..

[22] Du R., Huang C., Liu K., Li X., Dong Z. (2021). Targeting AURKA in Cancer: Molecular mechanisms and opportunities for Cancer therapy. Mol. Cancer.

[23] Kamenova S., Sharapkhanova A., Akimniyazova A., Kuzhybayeva K., Kondybayeva A., Rakhmetullina A., Pyrkova A., Ivashchenko A. (2022). piRNA and miRNA Can Suppress the Expression of Multiple Sclerosis Candidate Genes. Nanomaterials.

[24] Akimniyazova A.N., Niyazova T.K., Yurikova O.Y., Pyrkova A.Y., Zhanuzakov M.A., Ivashchenko A.T. (2022). piRNAs may regulate expression of candidate genes of esophageal adenocarcinoma. Front. Genet..

[25] Akimniyazova A., Yurikova O., Pyrkova A., Rakhmetullina A., Niyazova T., Ryskulova A.G., Ivashchenko A. (2022). In Silico Study of piRNA Interactions with the SARS-CoV-2 Genome. Int. J. Mol. Sci..

[26] Ameli Mojarad M., Ameli Mojarad M., Shojaee B., Nazemalhosseini-Mojarad E. (2022). piRNA: A promising biomarker in early detection of gastrointestinal cancer. Pathol. Res. Pract..

[27] Jian Z., Han Y., Li H. (2022). Potential roles of PIWI-interacting RNAs in lung cancer. Front. Oncol..

[28] Dabi Y., Bendifallah S., Suisse S., Haury J., Touboul C., Puchar A., Favier A., Daraï E. (2022). Overview of non-coding RNAs in breast cancers. Transl. Oncol..

[29] AmeliMojarad M., AmeliMojarad M., Wang J. (2022). The function of novel small non-coding RNAs (piRNAs, tRFs) and PIWI protein in colorectal cancer. Cancer Treat. Res. Commun..

[30] Goh T.X., Tan S.L., Roebuck M.M., Teo S.H., Kamarul T. (2022). A Systematic Review of Extracellular Vesicle-Derived Piwi-Interacting RNA in Human Body Fluid and Its Role in Disease Progression. Tissue Eng. Part C Methods.

[31] Guan S., Zhang Z., Wu J. (2022). Non-coding RNA delivery for bone tissue engineering: Progress, challenges, and potential solutions. iScience.

[32] Sun J., Yang X., Wang T., Xing Y., Chen H., Zhu S., Zeng J., Zhou Q., Chen F., Zhang X. (2022). Evaluating the Effects of Storage Conditions on Multiple Cell-Free RNAs in Plasma by High-Throughput Sequencing. Biopreserv. Biobank..

[33] Cho O., Kim D.W., Cheong J.Y. (2021). Screening Plasma Exosomal RNAs as Diagnostic Markers for Cervical Cancer: An Analysis of Patients Who Underwent Primary Chemoradiotherapy. Biomolecules.

[34] Wang J., Shi Y., Zhou H., Zhang P., Song T., Ying Z., Yu H., Li Y., Zhao Y., Zeng X. (2021). piRBase: Integrating piRNA annotation in all aspects. Nucleic Acids Res..

[35] Ivashchenko A., Berillo O., Pyrkova A., Niyazova R., Atambayeva S. (2014). MiR-3960 binding sites with mRNA of human genes. Bioinformation.

[36] Friedman R.A., Honig B.A. (1995). Free Energy Analysis of Nucleic Acid Base Stacking in Aqueous Solution. Biophys. J..

[37] Garg A., Heinemann U.A. (2018). Novel Form of RNA Double Helix Based on G·U and C·A+ Wobble Base Pairing. RNA.

[38] Leontis N.B., Stombaugh J., Westhof E. (2002). The Non-watson-crick Base Pairs and Their Associated Isostericity Matrices. Nucleic Acids Res..

[39] Davis E., Caiment F., Tordoir X., Cavaillé J., Ferguson-Smith A., Cockett N., Georges M., Charlier C. (2005). RNAi-Mediated Allelic Trans-interaction at the Imprinted Rtl1/Peg11 Locus. Curr. Biol..

