# Supplementary material for: Interactions of piRNAs with the mRNA of Candidate Genes in Esophageal Squamous Cell Carcinoma
Source: Curr Issues Mol Biol. 2023 Jul 23;45(7):6140–53. doi: 10.3390/cimb45070387 (PMC10378052; doi:10.3390/cimb45070387)
Supplement: Supplementary file 1 [file cimb-45-00387-s001.zip › Supplement 2/Table S1.pdf]

Table S1. List of candidate genes for ESCC.

Gene	PubMed	Gene	PubMed	Gene	PubMed
ABCG2	22236447	*EMP1*	22888304	*MMP13*	32035950
ADAMTS12	31236983	*ERCC1*	32611208	*MMP2*	32436041
ADAMTS2	29358879	*FSCN1*	35401239	*MMP3*	30969151
ADH1A	29248712	*GCOM1*	25823933	*MMP7*	21455340
ADH1B	27038040	*HLA-G*	29067119	*MMP9*	33572115
ALDH2	35788888	*HOXA10*	31950785	*MTHFR*	28046029
AURKA	32572158	*HPGD*	35317779	*NAT2*	25886288
BMI1	32239639	*IBSP*	31709184	*PDGFRA*	29358879
BMP7	23504348	*IGFBP3*	35782901	*PLAU*	33574243
C2orf40	27698864	*ITGA3*	35782901	*POSTN*	32613365
CCNA2	34737951	*ITGB4*	30479571	*PPP1R3C*	29844815
CCND1	34611831	*LAMA3*	29358879	*SASH1*	30443973
CCNE1	28800315	*LAMB3*	36415640	*SH3BGRL2*	25823933
COL10A1	35117161	*LAMC2*	29511340	*SIX1*	28260921
COL11A1	34790783	*MEIS1*	32819319	*SIX4*	33481352
COL1A1	35117161	*MFAP2*	29358879	*SOX2*	31991109
COL27A1	29358879	*MGLL*	25823933	*SPP1*	36467891
COL7A1	18331784	*MLF1*	29358879	*SULT1A1*	12455060
CYP1A1	26455829	*MMP1*	27130665	*SULT1A1*	12455060
EGR1	29047233	*MMP10*	22121946	*TCF3*	29358879
ELN	25823933	*MMP11*	29358879	*TP53*	34611831
EMP1	22888304	*MMP12*	35265129		

Note. Nine candidate piRNA target genes for esophageal squamous cell carcinoma are highlighted in yellow.
