# Supplementary material for: Interactions of piRNAs with the mRNA of Candidate Genes in Esophageal Squamous Cell Carcinoma
Source: Curr Issues Mol Biol. 2023 Jul 23;45(7):6140–53. doi: 10.3390/cimb45070387 (PMC10378052; doi:10.3390/cimb45070387)
Supplement: Supplementary file 1 [file cimb-45-00387-s001.zip › Supplement 2/Table S11.pdf]

Table S11. piRNAs that bind to the mRNA of *AURKA*, *ERCC1*, *SASH1* and *SIX4* genes.

piRNA	*AURKA*	*ERCC1*	*SASH1*	*SIX4*
piR-74077	+	+	+	+
piR-108578	+	+	+	+
piR-88365	+	+	-	+
piR-115026	+	-	-	+
piR-120925	+	-	+	+
piR-216660	+	+	+	-
piR-81461	+	-	+	-
piR-110734	+	+	+	+
piR-206181	+	+	+	-
piR-224694	+	+	+	-
piR-49581	+	+	+	-
piR-120170	+	+	+	+
piR-128529	+	+	+	-
piR-145556	+	-	+	+
piR-69165	+	+	-	+
piR-123162	+	+	-	+
piR-321692	+	-	-	+
piR-112665	+	-	+	+
piR-270870	+	+	-	+
piR-302301	+	-	-	+
piR-274756	+	-	-	+
piR-288286	+	+	+	+
piR-113908	+	+	-	+
piR-258342	+	+	-	-
piR-226914	+	+	-	-
piR-230823	+	+	-	-
piR-287550	+	+	-	-
piR-230473	+	+	+	-
piR-199338	+	-	+	-
piR-57622	+	+	+	-
piR-99677	+	+	+	+
piR-257600	+	+	+	-
piR-272067	+	+	+	-
piR-445186	+	+	+	-
piR-454666	+	+	+	-
piR-219325	+	+	+	+
piR-249982	+	+	+	+
piR-282903	+	+	+	+
piR-341211	+	-	+	+
piR-38326	+	+	+	+
piR-197774	+	-	+	+
piR-320670	+	+	-	+
piR-363930	+	-	+	-
piR-424861	+	+	+	+
piR-479539	+	-	-	+
piR-196512	+	-	+	+
piR-347859	+	-	+	+
piR-98281	+	-	-	+
piR-121234	+	+	+	-
piR-325698	+	+	+	+
piR-200086	+	+	-	+
piR-67883	+	+	+	+
piR-306330	+	+	-	+
piR-200135	+	+	+	+
piR-344301	+	-	-	+
piR-267379	+	+	+	+
Note. “+” indicates the presence of a piRNA BSs				
